# Associations between physical activity, mental health concerns, eating disorder symptoms, and emotional intelligence in adolescent athletes transitioning from COVID-19

**DOI:** 10.1186/s40337-023-00961-2

**Published:** 2024-01-02

**Authors:** Morteza Homayounnia Firouzjah, Heather Hower, Shahnaz Shahrbanian

**Affiliations:** 1 Department of Physical Education, Farhangian University, P.O. Box 14665-889, Tehran, Iran; 2 Department of Psychiatry, Eating Disorders Center for Treatment and Research, University of California at San Diego School of Medicine, 4510 Executive Drive, San Diego, CA 92121 USA; 3 Department of Sport Sciences, Faculty of Humanities, Tarbiat Modares University, Tehran, Iran

**Keywords:** Physical activity, Mental health, Eating disorders, Emotional intelligence, Adolescent athletes, Post COVID-19 pandemic

## Abstract

**Background:**

It is well known that COVID-19 significantly disrupted the routines of school sports for adolescent athletes. In transitioning from this “change event,” athletes may need support with resuming their pre-pandemic level of activities, and addressing their ongoing mental health concerns, including exacerbated eating disorder symptoms. Emotional intelligence (the ability to understand emotions, influencing decisions and motivation) is a factor that has not yet been studied during this transition, but may serve as a coping mechanism for altered physical activity, mental health, and eating disorder symptoms.

**Methods:**

Participants of the study included 315 Iranian adolescent athletes who transitioned back to 1 of 10 sports post quarantine restrictions (January 2022–January 2023). Physical activity and related stages of motivation for behavioral change were assessed by the Physical Activity Stages of Change Questionnaire, mental health concerns were assessed by the General Health Questionnaire-28, eating disorder symptoms were assessed by the Eating Attitudes Test-26, and emotional intelligence was assessed by the Schutte Self-Report Emotional Intelligence Test.

**Results:**

A three-way Multivariate Analysis of Variance (MANOVA) was conducted in order to test the overall differences between the 5 physical activity and behavioral change motivation groups (Pre-Contemplation, Contemplation, Preparation, Action, and Maintenance) on each of the 3 health measures (mental health concerns, eating disorder symptoms, and emotional intelligence); significant main effects were found for each measure. Fischer’s Least Significant Difference Post-Hoc Test indicated that there were significant differences between the Pre-Contemplation and Preparation groups, as well as the Preparation and Action groups, on all of the health measure mean variable scores, which contributed to the MANOVA significant main effects.

**Conclusions:**

Participants in the Pre-Contemplation group (not intending to make behavioral changes within the next 6 months) had less mental health, higher eating pathology, and lower emotional intelligence, compared to participants who were in the Preparation group (intending to make behavioral changes within the next 1 month). Similarly, participants in the Preparation group had less mental health, higher eating pathology, and lower emotional intelligence, compared to participants who were in the Action group (has made specific, overt behavioral changes within the preceding 6 months). Overall, the findings from the current study highlight the need for sports professionals working with adolescent athletes transitioning from COVID-19 to monitor these aspects of mental, eating, and emotional health. Given that those in earlier motivation stages had more health concerns compared to those in later stages, professionals should encourage progression from the Pre-Contemplation to Action/Maintenance stage in order to improve health outcomes.

**Supplementary Information:**

The online version contains supplementary material available at 10.1186/s40337-023-00961-2.

## Abstract (plain language summary)

It is well known that COVID-19 significantly disrupted the routines of school sports for adolescent athletes. In transitioning from this “change event,” athletes may need support with resuming their pre-pandemic level of activities, and addressing their ongoing mental health concerns, including exacerbated eating disorder symptoms. Emotional intelligence (the ability to understand emotions, influencing decisions and motivation) is a factor that has not yet been studied during this transition, but may serve as a coping mechanism for altered physical activity, mental health, and eating disorder symptoms. Participants of the study included 315 Iranian adolescent athletes who transitioned back to 1 of 10 sports post quarantine restrictions (January 2022–January 2023). Those who did not intend to make changes to their behaviors within the next 6 months (in the pre-contemplation motivation stage) had less mental health, higher eating pathology, and lower emotional intelligence, compared to those who were intending to make behavioral changes within the next 1 month (in the preparation motivation stage). Similarly, those who were in the preparation motivation stage had less mental health, higher eating pathology, and lower emotional intelligence, compared to shoes who had made specific, observable behavioral changes within the preceding 6 months (in the action motivation stage). Overall, the findings from the current study highlight the need for sports professionals working with adolescent athletes transitioning from COVID-19 to monitor these aspects of mental, eating, and emotional health. Given that those in earlier motivation stages had more health concerns compared to those in later stages, professionals should encourage progression from the Pre-Contemplation to Action/Maintenance stage in order to improve health outcomes.

**Supplementary Information:**

The online version contains supplementary material available at 10.1186/s40337-023-00961-2.

## Background

It is well known that COVID-19 significantly interrupted the routines of school and organized sports for adolescents (e.g., cancellation of in-person training activities, public sporting events), which often led to decreased physical activity without access to exercise facilities [1]. The pandemic can be viewed as a “change-event” that disrupted the quality and intensity of athletic engagement [2]. Some of the beneficial physical adaptations in the body as a result of a regimented training program (e.g., increases in blood and blood plasma volume, cardiac output and stroke volume during maximal efforts, muscular hypertrophy) [3] have been shown to reverse with deconditioning (e.g., extended periods of lowered or no activity) [4]. Decreased physical activity, particularly within the context of unpredicted and uncontrolled “change-events,” may have negative impacts (e.g., injuries) [5] that could have occurred.

For some athletes who continued to play their sport during the beginning of the pandemic until their country imposed a lockdown, they experienced anticipatory anxiety about potentially contracting COVID-19, which affected their focus and athletic functioning. Indeed, in a study conducted by Yidiz et al. (October 2021) in Turkey, 972 competitive adolescent-adult athletes (both men and women, age range 17–35 years) reported significant concerns about contracting the virus which negatively impacted their ability to play their sport [6].

After country restrictions on athletic play were established, the element of social isolation during the quarantine provided additional mental [7, 8] challenges to athletes’ ability to maintain their usual healthy performance levels [9]. In a study conducted mid-way through the pandemic (June 2021), Denerel and colleagues assessed the effects of home isolation thus far on the mental health of adolescent athletes in Australia [8]. Participants included 940 team athletes, 274 individual athletes, and 131 nonathlete controls (both boys and girls, age range 12–17 years); they reported that 88% of the athletes did not meet the physical activity recommendations for adolescents, and 42.8% felt depressed at the time [8]. In a follow-up study with a subsample of the Yidiz et al. cohort (256 competitive athletes) during the lockdown in Turkey (January 2022), they found that an increase in the athletes’ depression or anxiety levels may in turn cause an increase in the other disorder’s levels [10]. Overall, the negative effects of the COVID-19 lockdown on mental health have been widely recognized and acknowledged, for the general population, as well as for athletes [1, 2, 7, 9–12].

Specific mental health concerns centering on a surge in eating disorder symptoms (especially relevant for athletes, who need to fuel their bodies for optimal athletic performance) was reported early in the COVID-19 pandemic (April–May 2020) among current and former athletes by Buckley et al. [12]. In a convergent mixed-methods design with 204 adult athletes (41 different individual and team sports, from club through elite athlete levels), they reported that there was a significant difference in ED scores (e.g., inhibitory food control, binge eating) between those who perceived their relationship with food to have gotten worse during COVID-19, vs. those who perceived it stayed the same (χ^2^(2,198) = 15.5, *p* = 0.000) [12].

During this time of distress, a “decisional balance” must be made (weighing the “pros” vs. the “cons”) as to whether to alter behaviors to improve health. In order to assess this, Marcus and colleagues developed a scale (Physical Activity Stages of Change Questionnaire; PASCQ) [13] which deliberately maps onto Prochaska’s transtheoretical model of behavioral change [14]. Within this framework, individuals’ perception of the positive benefits (“pros”) are balanced against the negative obstacles (“cons”), which aids in decision-making [15]. According to this model, behavioral change is a process that unfolds over time, and involves progress through a series of five stages: (1) Pre-Contemplation (the individual is not intending to take action within the next 6 months, e.g., the athlete is not considering changing their current level of athletic activity, whether it be relatively low or high); (2) Contemplation (the individual is intending to take action within the next 6 months, e.g., the athlete is considering changing their current level of athletic activity within the next 6 months, either to increase it, or decrease it); (3) Preparation (the individual is intending to take action within the next 1 month, e.g., the athlete is intending to either increase or decrease their current level of athletic activity within the next 1 month); (4) Action (the individual has made specific, overt modifications in their behavior within the preceding 6 months; e.g., the athlete has made observable changes in either increasing or decreasing their level of athletic activity within the past 6 months); and (5) Maintenance (the individual is working to prevent relapse into old behaviors, but does not need to apply change processes as frequently as in the action stage, e.g., the athlete is in a routine of athletic activity that has stabilized at their desired level, and less motivation is needed to improve this level) [16].

What is acknowledged, though, is how difficult it is to change behaviors; specifically, how challenging it is to increase activity levels [13]. Indeed, Marcus et al. examined exercise adoption and maintenance, and reported four patterns of stage change: (1) “adopters” (those who become more active over time, 26%); (2) “relapsers” (those who become less active over time, 15%); (3) “stable sedentary” (those who did not change their level of non-activity, 32%); and (4) “stable active” (those who did not change their level of regular activity, 27%) [13]. Thus, the majority of participants (59%) in the Marcus et al. exercise study did not change their “stable” activity levels over the follow-up. However, to the best of our knowledge, these stages of activity change have not yet been applied to an athletic population, including during the very significant transitional “change event” from quarantine lockdown to having activity restrictions removed.

A factor that may influence motivation to change activity levels (and thus tip the “decisional balance” towards increasing/decreasing activity per the athlete’s personal goals), is the level of emotional intelligence (the ability to understand and regulate emotions in oneself and in others) [17] that the athlete possesses. Emotional intelligence involves a set of abilities (e.g., self-awareness, self-management, social awareness, communication management) [17] which can facilitate coping with difficult emotions in general (e.g., anxiety, depression, loneliness) [18].

More specifically, a meta-analysis by Zhang et al. [19] indicated that those with higher emotional intelligence are less likely to have eating disorders. Emotional intelligence was evaluated by Rubio and colleagues [20] in a sample of 165 students studying undergraduate and master’s degrees related to physical activity and sport sciences (mean age = 20.33 years, standard deviation [*SD*] = 3.44 years). They indicated that emotional intelligence predicted levels of anxiety, motivation, and leadership within an athletic context. Levine et al. [21] conducted a qualitative assessment with 20 student athletes (average age = 20 years, range = 18–23 years) on factors that affected their experiences as they transitioned back into their sports from COVID-19 (in between January-August 2021). They reported themes of wavering confidence and motivation (e.g., inability to train led to feeling less ready, uncertainty hurt motivation), increased stress and anxiety (e.g., social restrictions contributed to stress), but also use of adaptive coping strategies (e.g., social support from teammates, coaches, and family). Thus, it seems that emotional intelligence may serve as a coping mechanism for general mental health concerns, and specific eating disorder symptoms, and may also improve athletic performance. However, to the best of our knowledge, the relationships between these factors have not yet been assessed in athletes, particularly during a crucial transition from the COVID-19 pandemic.

### Aim of the present study

While the negative effects of the pandemic are well known for the general population [22–24], there remain gaps in the literature on athletes and their specific associations with stages of physical activity change, mental health, eating disorder symptoms, and emotional intelligence coping skills as they transition back into their sports. The aim of the present study was therefore to examine these associations between physical activity, mental health concerns, eating disorder symptoms, and emotional intelligence in male and female adolescent athletes in the transition from COVID-19 as the area quarantine restrictions were lifted (January 2022-January 2023).

## Methods

### Participants and procedures

The current study serves as a follow-up to a prior study by these authors [25] that was conducted at the beginning of the quarantine period (June–August 2020). The procedure for both of the studies’ recruitment, and inclusion and exclusion criteria, are detailed below.

In the previous study [25], participants (N = 124) residing in Mazandaran province, Iran, were recruited via a research coordinator who had communicated with the respective officials of the 6 sports groups that were assessed. Through the officials, the research coordinator contacted the adolescents and their parents via phone and email to gauge their interest in participating in the study (focused on the comparison of eating disorder symptoms and body image between individual and team sport male adolescent athletes during the lockdown). The study inclusion criterion was: (1) aged 12–19 years old (“adolescence,” as defined by Rice [26]). The study exclusion criterion was: (1) lack of regular physical activity, assessed via the WHO Global Physical Activity Questionnaire (WHO-GPAQ) self-report [27]. This criterion was included in order to differentiate between a lack of regular physical activity due to normal patterns (e.g., sedentary lifestyle), vs. a lack of regular physical activity due to changes during the COVID-19 pandemic assessment period (e.g., reduced exercise levels). For the lockdown period, all athletes were given a practice/playing schedule to maintain fitness at home, or in socially isolated environments. For some, this may have meant relatively lower physical activity than pre-pandemic levels (e.g., for team sports players, the coaches’ focus might be on maintaining individual athletes’ strength and flexibility, vs. running as much as they did during their pre-pandemic games, such as in the case with soccer). Thus, the athletes had continued exercise during the lockdown, albeit with modified routines and formats. The amount of regular physical activity was tracked by the adolescent athletes and their parents, and the accuracy of the level of physical activity was confirmed by the study researchers. De-identified data for this study (i.e., eating disorder symptoms and body image) were collected online at the participants’ homes (from June–August 2020) and reviewed by the study researchers. The study procedures were explained, and informed consent was obtained from all participants and their parents prior to study initiation. The governing Medical Ethics and History of Medicine Research Center for each Iranian university study site (Farhangian University, Islamic Azad University, and Tarbiat Modarres University; the coordinating site) reviewed and approved the study before enrollment of any participant. All methods were performed in accordance with the Declaration of Helsinki.

In the current (follow-up) study, participants (N = 315; male n = 270, female n = 93) residing in Mazandaran province were similarly recruited via a research coordinator who had communicated with the respective officials of the 10 sports groups that were assessed (Football, Volleyball, Taekwondo, Wrestling, Handball, Physical Fitness Training, Badminton, Ping Pong, and Running). The study sample was deliberately expanded from the prior one to include more participants (N = 124 vs. N = 315) of younger age (12 vs. 10), both sexes (the prior study only included males), and playing in more sports (6 vs. 10), during the transition period from COVID-19 as the area restrictions were lifted (social distancing was not required, and athletes were allowed to return to their sports; assessed from January 2022 to January 2023). The study inclusion criterion was: (1) aged 10 to 19 years old (“adolescence,” as defined by Rice [26], and by Gentry and Campbell [28]). The study exclusion criterion was: (1) lack of physical activity in the past 6 months, as assessed by the PASCQ [29] (see below for more details). This criterion was included in order to eliminate anyone who did not exercise during the past 6 months of quarantine, despite the at-home training program that the coaches designed for them (noted above), thus establishing a “baseline” of minimal activity from which to compare the other variables. The amount of regular physical activity was tracked by the adolescents, their parents, and coaches, and the accuracy of the level of physical activity was confirmed by the study researchers. Athletes and their parents met the research coordinator at their sports facility, where the study procedures were explained in person, and informed consent was obtained from all participants and their parents prior to study initiation. Of note, the adolescents’ comprehension of the consent and the self-report measures that were utilized (particularly for those who were younger in age) were assessed by the research coordinator and parents to ensure their understanding (e.g., the adolescents were able to repeat back the purpose of the study, and complete the self-reports). As with the prior study, the governing Medical Ethics and History of Medicine Research Center for each Iranian university study site reviewed and approved the study protocol and procedures before enrollment of any participant (Tarbiat Modarres University approval #IR.MODARES. REC.1399.097, granted on October 10, 2020, for study assessment January 2022-January 2023). All methods were performed in accordance with the Declaration of Helsinki.

### Measures

De-identified study data (distribution of male and female participants across the different sports modalities, self-report questionnaires) were collected in person at the participants’ sports’ facilities after the quarantine period of COVID-19. Each of the self-report questionnaires had been previously been translated from English into Persian (Farsi); they were found to be reliable and valid for the Iranian adolescent population (please see below for the psychometrics of each questionnaire). These Farsi versions were utilized in the current study for the participants’ comfort and familiarity with the language.

### Physical activity stages of change questionnaire (PASCQ)

The PASCQ [29] is a 4-item self-report measure of the stages of physical activity behavior change. The authors, Marcus and colleagues, define physical activity (or exercise) as “walking briskly, jogging, bicycling, swimming, or any other physical activity in which the exertion is at least as intense as these activities” [29]. For physical activity to be regular, it “must add up to a total of 30 min or more per day, and be done at least 5 days per week” [29]. The self-report items are: (1) I am currently physically active; (2) I intend to become more physically active in the next 6 months; (3) I currently engage in regular physical activity; and (4) I have been regularly physically active for the past 6 months. Of note, the amount of physical activity was tracked by the adolescent athletes, their parents, and coaches, and the accuracy of the level of physical activity was confirmed by the study researchers (e.g., review of sports practice, log books).

As mentioned above, the PASQ deliberately maps on to Prochaska’s transtheoretical model of behavioral change [14], in which the “pros” vs “cons” of change are weighed in a “decisional balance” [30] (e.g., whether to increase or decrease your current level of exercise). Again, the stages are: (1) Pre-Contemplation (physically inactive and do not intend to start exercising in the next 6 months); (2) Contemplation (physically inactive and plan to start exercising in the next 6 months); (3) Preparation (irregular physical activity and exercise less than 3 times a week, each time for 30 min); (4) Action (regular physical activity, but less than 6 months); and (5) Maintenance (regular physical activity for more than 6 months) [29]. PASCQ item responses are dichotomous (No/Yes), and categorized as: (1) Pre-Contemplation (Item 1 = No, Item 2 = No); (2) Contemplation (Item 1 = Yes, Item 2 = No); (3) Preparation (Item 1 = Yes, Item 2 = Yes, Item 3 = No); (4) Action (Item 1 = Yes, Item 2 = Yes, Item 3 = Yes, Item 4 = No); and (5) Maintenance (Item 1 = Yes, Item 2 = Yes, Item 3 = Yes, Item 4 = Yes).

Marcus and colleagues [29] reported that the PASCQ items (in the English version) have an excellent internal consistency reliability (Cronbach’s alpha α = 0.87), a good test–retest reliability (Spearman’s rho ρ = 0.78, *p* < 0.001), and an excellent concurrent validity with the Physical Activity Recall (PAR) Questionnaire [31] (Pearson’s product moment correlation *r* = 0.85, *p* < 0.01). The Persian (Farsi) version of the PASQ (previously translated and validated in an Iranian adolescent population by Nezami et al. in 2020) [32] was utilized in the current study. The present sample had an excellent internal consistency reliability (Cronbach's alpha α = 0.92), and an excellent criterion validity (Pearson’s product moment correlation *r* = 0.86,* p* < 0.01).

### General health questionnaire-28 (GHQ-28)

The GHQ-28 [33, 34] is a 28-item self-report measure of emotional distress (mental health concerns) with 4 subscales: (1) somatic symptoms; (2) anxiety/insomnia; (3) social dysfunction; and (4) severe depression. Response agreement is rated on a 4-point Likert scale (0 = “Not at all,” to 3 = “Much more than usual”), with total scores ranging from 0 to 84 (clinical cut-off total score of 23/24 is the threshold for the presence of emotional distress).

The author, Goldberg, and other investigators, reported that the GHQ-28 items (in the English version) have excellent reliability overall; internal consistency reliability (Cronbach’s α = 0.93) [35, 36]; split-half reliability (Cronbach’s α = 0.95) [35]; inter-rater and intra-rater reliability (Cronbach’s α 0.9–0.95) [36]; and test–retest reliability (Spearman’s rho ρ = 0.93, *p* < 0.01) [37]. Validity for the GHQ-28 (in the English version) has also been reported to be excellent overall; GHQ-28 subscales criterion validity (Pearson’s product moment correlation *r* = 0.73–96,* p* < 0.01) [30]; and concurrent validity with the Clinical Interview Schedule (CIS) (Pearson’s product moment correlation *r* = 0.83, *p* < 0.01) [38, 39]. The Persian (Farsi) version of the GHQ-28 (previously translated and validated in an Iranian adolescent population by Molavi in 2002) was utilized in the current study [40]. The present sample had an excellent wholescale GHQ-28 internal consistency reliability (Cronbach’s α = 0.96), and a good–excellent split-half reliability for the GHQ-28 subscales (Cronbach’s α = 0.73–0.96).

### Eating Attitudes Test-26 (EAT-26)

The EAT-26 [41] is a 26-item self-report measure of thoughts and behaviors related to eating disorder symptoms with 3 subscales: (1) thoughts and behaviors related to dieting (Dieting); (2) preoccupation with food and impulses to binge and purge (Bulimia and Food Preoccupation); and (3) attempts to control food intake (Oral Control). Response agreement is rated on a 4-point Likert scale (0 = “Never,” to 3 = “Always”), with total scores ranging from 0 to 78 (a clinical cut-off score of ≥ 20 indicates that the person scores within the clinical level; as high as a person with an eating disorder diagnosis, but they may not have an official diagnosis). The authors, Garner et al., reported that the EAT-26 items (in the English version) have an excellent internal consistency reliability (Cronbach’s alpha α = 0.88), and excellent content validity (wholescale EAT-26 Pearson’s product moment correlation* r* = 0.91, *p* < 0.01); EAT-26 subscales Pearson’s product moment correlation *r* = 0.78, *p* < 0.01) [41].

The wholescale EAT-26 items (in the English version) in general populations and patient samples have been shown to have excellent overall reliability (internal consistency reliability Cronbach’s alpha α = 0.91; test–retest reliability Spearman’s rho ρ = 0.98, *p* < 0.01), and validity (criterion validity Pearson’s product moment correlation *r* = 0.90, *p* < 0.01) [42–44]. The Persian (Farsi) version of the EAT-26, including the clinical cut-off score of ≥ 20 indicating disordered eating (previously translated and validated in an Iranian adolescent population by Sanaei et al. in [45], was utilized in the current study. The present sample had an excellent wholescale EAT-26 internal consistency reliability (Cronbach's alpha α = 0.90), and a good test–retest reliability for the EAT-26 subscales (Spearman’s rho ρ = 0.84–0.89, *p* < 0.001).

### Schutte Self-Report Emotional Intelligence Test (SSEIT)

The SSEIT [46] is a 33-item self-report measure of emotional intelligence with 3 subscales: (1) evaluation and expression of emotion; (2) management and regulation of emotion; and (3) application of emotion. Response agreement is rated on a 5-point Likert scale (1 = “strongly agree,” to 5 = “strongly disagree”), with total scores ranging from 33–165. The higher the score on this assessment, the higher the level of emotional intelligence (there is no clinical cut-off score). The authors, Schutte and colleagues, reported that the SSEIT items (in the English version) have an excellent internal consistency reliability (Cronbach’s alpha α = 0.90), and good predictive validity (Pearson’s product moment correlation *r* = 0.32, *p* < 0.01) [46]. The Persian (Farsi) version of the SSEIT (previously translated and validated in an Iranian adolescent population by Ashouri et al. in 2019) was utilized in the current study [47].The present sample had a good wholescale SSEIT internal consistency reliability (Cronbach’s alpha α =  > 0.79), and a good internal consistency for the SSEIT subscales (Cronbach’s alpha α =  > 0.73-0.0.78).

### Data analyses

All data were entered into the Statistical Package for Social Science (SPSS) 24 software for analyses [48]. The distribution of male and female adolescent athlete participants across the different sports modalities was assessed (sex, age, and sport type) (see Table 1). Pearson’s correlation coefficients among physical activity, mental health concerns, eating disorder symptoms, and emotional intelligence were calculated (see Table 2). Participants were grouped into one of the 5 stages of physical activity and behavioral change motivation based upon their response to the PASCQ: (1) Pre-Contemplation; (2) Contemplation; (3) Preparation; (4) Action; or (5) Maintenance. The means and standard deviations *(SD)* of the participant scores on the 3 health measure variables by the 5 groups were determined: (1) mental health concerns (GHQ-28); (2) eating disorder symptoms (EAT-26); and (3) emotional intelligence (SSEIT) (see Table 3). The frequency and percentage of participants who scored above the clinical cut-offs (≥ 20) on the three EAT-26 subscales for disordered eating (Dieting, Bulimia and Food Preoccupation, and Oral Control), as well as those who scored below the clinical cut-offs, were analyzed by each of the 5 groups (see Additional file 1: Table S1). A three-way Multivariate Analysis of Variance (MANOVA) [49] was conducted in order to test the overall differences between the 5 groups on each of the 3 health measures (see Table 3, and Additional file 2: Table S2). Given that there were significant differences in the MANOVA main effects, Fischer’s Least Significant Difference (LSD) Post-Hoc Test was used to investigate exactly which of the 5 groups had statistically significant health measure mean variable score differences between them (see Table 4).

**Table 1:** Distribution of male and female participants across the different sports modalities

Sport Sex	Football	Volleyball	Basketball	Tae Kwon Do	Wrestling	Handball	Physical fitness training	Badminton	Ping Pong	Running
Male (%)	25 (18.38%)	14 (8.58%)	13 (7.97%)	14 (8.58%)	17 (10.42%)	15 (9.20%)	5 (3.06%)	2 (1.22%)	3 (1.84%)	3 (1.84%)
Female (%)	8 (4.90%)	10 (6.13%)	8 (4.90%)	7 (4.29%)	0	5 (3.06%)	3 (1.84%)	4 (2.45%)	4 (2.45%)	3 (1.84%)
Total (%)	33 (20.24%)	24 (14.74%)	21 (12.88%)	21 (12.88%)	17 (10.42%)	20 (12.26%)	8 (4.90%)	6 (3.68%)	7 (4.29%)	6 (3.68%)

**Table 2:** Correlations among physical activity, mental health concerns, eating disorder symptoms, and emotional intelligence

	Physical activity	Mental health concerns	Eating disorder symptoms	Emotional intelligence
Physical activity	1.00	0.63**	0.25**	0.36**
Mental health concerns	0.63**	1.00	− 0.33**	0.35**
Eating disorder symptoms	0.25**	− 0.33**	1.00	− 0.21**
Emotional intelligence	0.36**	0.35**	− 0.21**	1.00

**p < 0.01

Physical activity and behavioral change motivation was assessed by the Physical Activity Stages of Change Questionnaire (PASCQ) [29]; Mental health concerns were assessed by the General Health Questionnaire (GHQ-28) [50]; Eating disorder symptoms were assessed by the Eating Attitudes Test-26 (EAT-26) [41]; Emotional intelligence was assessed by the Schutte Emotional Intelligence Questionnaire (SSEIT) [46]

**Table 3:** Comparison between groups

Physical activity stage	Pre-contemplation (n = 97, 30.8%)		Contemplation (n = 67, 21.3%)		Preparation (n = 54, 17.1%)		Action (n = 28, 8.9%)		Maintenance (n = 69, 21.9%)		MANOVA (F)
Measures	Mean	SD	Mean	SD	Mean	SD	Mean	SD	Mean	SD	
Mental health	3.54	16.64	2.47	17.52	3.45	17.19	2.97	19.64	3.71	18.68	10.00**
Eating disorder symptoms	6.85	14.47	5.96	16.78	6.47	18.83	6.81	21.43	7.47	23.57	11.66**
Emotional intelligence	1.17	7.29	1.85	8.49	1.37	7.67	1.54	8.71	1.38	8.47	7.88**

**p < 0.01

*MANOVA* = Multiple Analysis of Variance [49]; *SD* = Standard Deviation; Physical activity and behavioral change motivation was assessed by the Physical Activity Stages of Change Questionnaire (PASCQ) [29]; Mental health concerns were assessed by the General Health Questionnaire (GHQ-28) [50]; Eating disorder symptoms were assessed by the Eating Attitudes Test-26 (EAT-26) [41]; Emotional intelligence was assessed by the Schutte Emotional Intelligence Questionnaire (SSEIT) [46]

**Table 4:** Significant differences between groups

Measures	Mental health concerns		Eating disorder symptoms		Emotional intelligence	
Physical activity stage	Mean difference	*p*-value	Mean difference	*p*-value	Mean difference	*p*-value
Pre-contemplation-contemplation	8.64	0.061	− 4.22	0.012*	6.51	0.043*
Pre-contemplation-preparation	8.46	0.028*	− 3.54	0.042*	3.81	0.025*
Pre-contemplation-action	5.39	0.035*	− 3.68	0.022*	− 5.39	0.237
Pre-contemplation-maintenance	6.48	0.051	− 4.65	0.035*	− 4.36	0.046*
Contemplation-preparation	− 7.54	0.128	− 2.54	0.128	− 4.62	0.491
Contemplation-action	− 6.35	0.194	− 3.64	0.524	6.52	0.062
Contemplation-maintenance	5.67	0.253	− 2.73	0.063	5.58	0.035*
Preparation-action	5.92	0.028*	− 4.92	0.046*	6.71	0.039*
Preparation-Maintenance	4.65	0.094	− 3.54	0.037*	5.91	0.071
Action-maintenance	6.82	0.047*	− 2.35	0.064	4.83	0.028*

*p < 0.05

## Results

### Distribution of male and female participants across the different sports modalities

Of the eligible participants who were contacted during the transition from COVID-19 as the area restrictions were lifted from January 2022 through January 2023 (N = 947), N = 426 agreed/consented to participate, and N = 315 completed the self-report measures.

The distribution of male and female participants across the different sports modalities are presented in Table 1. In summary, the sample was comprised of N = 315 adolescent athletes (male sex n = 212, female sex n = 103), whose average age was 16 years old (*SD* ± 2.56 years old, range = 10–19 years old).

### Correlations among physical activity, mental health concerns, eating disorder symptoms, and emotional intelligence

Pearson’s correlation coefficients among physical activity, mental health concerns, eating disorder symptoms, and emotional intelligence were calculated and are noted in Table 2. In summary, all of the study measures were significantly correlated with each other, indicating that they each had an impact on the other. Higher physical activity was associated with greater mental health, while higher eating pathology was associated with less mental health. Nevertheless, higher emotional intelligence was associated with greater mental health, as well as lower eating pathology.

### Comparisons between groups

Participants were grouped into one of the 5 stages of physical activity and behavioral change motivation based upon their response to the PASCQ: (1) Pre-Contemplation (n = 97); (2) Contemplation (n = 67); (3) Preparation (n = 54); (4) Action (n = 28); or (5) Maintenance (n = 69). The means and *SD* of the 3 health measures (mental health concerns, eating disorder symptoms, and emotional intelligence variables) by the 5 groups are presented in Table 3. In summary, per Marcus et al.’s definition of “stable” [13] (physical activity levels, whether relatively low or high, have remained unchanged in the past 6 months), the majority of participants fell into this category while transitioning from COVID-19 lockdown. Most did not consider changing their physical activity levels in the past 6 months (again, which occurs when people do not feel that any changes need to be made-Pre-Contemplation; n = 97; 30.8%), followed by those who were maintaining their physical activity levels in the past 6 months (which occurs when people recognize that the changes that they previously made were beneficial, and they are working to continue them-Maintenance; n = 69; 21.9%). Participants in the Maintenance group had significantly higher mean scores on the 3 health measures compared to participants in the Pre-Contemplation group; mental health concerns (mean = 3.71, *SD* = 18.68 vs. mean = 3.54, *SD* = 16.64), eating disorder symptoms (mean = 7.47, *SD* = 23.75 vs. mean = 6.85, *SD* = 14.47), and emotional intelligence (mean = 1.38, *SD* = 8.47 vs. mean = 1.17, *SD* = 7.29). In other words, participants in the Maintenance group (who maintained their physical activity levels in the past 6 months) reported higher scores on mental health concerns (indicating better mental health status) and emotional intelligence (indicating better coping skills), but also increased eating disorder symptoms, compared to participants in the Pre-Contemplation group (who did not consider changing their physical activity levels in the past 6 months).

The frequency and percentage of participants who scored above the clinical cut-offs (≥ 20) on the three EAT-26 subscales for disordered eating (Dieting, Bulimia and Food Preoccupation, Oral Control), as well as those who scored below the clinical cut-offs, were analyzed by each of the 5 groups, and the findings are noted in Additional file 1: Table S1. In summary, the majority of participants (n = 163; 51.74%) who scored above the clinical cut-offs were in the Pre-Contemplation stage compared to the other stages. In other words, most participants above the clinical cut-off did not intend to change their behaviors related to eating (dieting, preoccupation with food, impulses to binge and purge, attempts to control food intake) within the next 6 months (again, Pre-Contemplation occurs when people do not feel that any changes need to me made).

A three-way Multivariate Analysis of Variance (MANOVA) [49] was conducted in order to test the overall differences between the 5 groups on each of the 3 health measures. The F and *p* values are noted in Table 3, and the full MANOVA is presented in Additional file 2: Table S2. In summary, there were significant main effects for the MANOVA on each of the 3 health measures, indicating that there are some statistically significant differences between the groups.

Fischer’s LSD Post-Hoc Test was used to investigate exactly which of the 5 groups had the statistically significant health measure mean variable score differences between them that contributed to the MANOVA significant main effects, and the findings are detailed in Table 4. In summary, there was a significant difference between the Pre-Contemplation and Contemplation groups on eating disorder symptoms and emotional intelligence. There was a significant difference between the Pre-Contemplation and Preparation groups on mental health concerns, eating disorder symptoms, and emotional intelligence. There was a significant difference between the Pre-Contemplation and Action groups on mental health concerns and eating disorder symptoms. There was a significant difference between the Pre-Contemplation and Maintenance groups on eating disorder symptoms and emotional intelligence. There was a significant difference between the Contemplation and Maintenance groups on emotional intelligence. There was a significant difference between the Preparation and Action groups on mental health concerns, eating disorder symptoms, and emotional intelligence. There was a significant difference between the Preparation and Maintenance groups on eating disorder symptoms. There was a significant difference between the Action and Maintenance groups on mental health concerns and emotional intelligence. Of note, there were significant differences in particular in between the Pre-Contemplation and Preparation groups, as well as the Preparation and Action groups, on all of the health measure mean variable scores, which contributed to the MANOVA significant main effects. In other words, participants in the Pre-Contemplation group had less mental health, higher eating pathology, and lower emotional intelligence, compared to participants who were in the Preparation group. Similarly, participants in the Preparation group had less mental health, higher eating pathology, and lower emotional intelligence, compared to participants who were in the Action group. Thus, participants who were in an earlier motivation stage had more health concerns compared to participants who were in a later motivation stage (Pre-Contemplation vs. Preparation, Preparation vs. Action).

## Discussion

As noted above, while the negative effects of the pandemic are well known for the general population [22–24], there were gaps in the literature on athletes and their specific associations with physical activity and related stages of motivation for behavioral change, mental health concerns, eating disorder symptoms, and emotional intelligence coping skills as they transition back into their sports. The aim of the present study was therefore to examine these associations between physical activity, mental health concerns, eating disorder symptoms, and emotional intelligence in male and female adolescent athletes in the transition from COVID-19 as the area quarantine restrictions were lifted (January 2022–January 2023). To the best of our knowledge, this study provides novel findings specifically on physical activity and related stages of motivation for behavioral change, as well as emotional intelligence coping skills, in an athletic population, as well as their interactions with the more established factors of mental health concerns and eating disorder symptoms, during this very significant transitional “change event.”

In summarizing the findings, overall, all of the study measures were significantly correlated with each other, indicating that they each had an impact on the other. Higher physical activity was associated with greater mental health, while higher eating pathology was associated with less mental health. Nevertheless, higher emotional intelligence was associated with greater mental health, as well as less eating pathology.

Focusing on physical activity, participants were grouped into one of the 5 stages of physical activity and behavioral change motivation: (1) Pre-Contemplation (the individual is not intending to take action within the next 6 months); (2) Contemplation (the individual is intending to take action within the next 6 months); (3) Preparation (the individual is intending to take action within the next 1 month); (4) Action (the individual has made specific, overt modifications in their behavior within the preceding 6 months); and (5) Maintenance (the individual is working to prevent relapse into old behaviors, but does not need to apply change processes as frequently as in the action stage). Per Marcus et al.’s definition of “stable” [13] (physical activity levels, whether relatively low or high, have remained unchanged in the past 6 months), the majority of participants fell into this category while transitioning from COVID-19 lockdown. Most were in the Pre-Contemplation physical activity and behavioral change motivation group, followed by those who were in the Maintenance group. Participants in the Maintenance group had significantly higher mean scores on the 3 health measures compared to participants in the Pre-Contemplation group. In other words, participants who maintained previous physical activity level changes reported higher scores on mental health concerns (indicating better mental health status) and emotional intelligence (indicating better coping skills), but also increased eating disorder symptoms, compared to participants in the Pre-Contemplation group.

In regard to eating disorder symptoms, the majority of participants who scored above the clinical cut-offs for the three subscale measures were in the Pre-Contemplation group compared to the other groups. In other words, most participants above the clinical cut-off did not intend to change their behaviors related to eating (dieting, preoccupation with food, impulses to binge and purge, attempts to control food intake) within the next 6 months (again, Pre-Contemplation occurs when people do not feel that any changes need to me made).

Examining the relationship between all of the study variables further, a MANOVA indicated that there were overall significant differences between the 5 groups on each of the 3 health measures (*p* < 0.01) Fischer’s LSD Post-Hoc Test investigated exactly which of the 5 groups had the statistically significant health measure mean variable score differences between them (*p* < 0.05). Of note, there were significant differences between the Pre-Contemplation and Preparation groups, as well as the Preparation and Action groups, on all of the health measure mean variable scores, which contributed to the MANOVA significant main effects. In other words, participants in the Pre-Contemplation group had less mental health, higher eating pathology, and lower emotional intelligence, compared to participants who were in the Preparation group. Similarly, participants in the Preparation group had less mental health, higher eating pathology, and lower emotional intelligence, compared to participants who were in the Action group. Thus, participants who were in an earlier motivation stage had more health concerns compared to participants who were in a later motivation stage (Pre-Contemplation vs. Preparation, Preparation vs. Action).

As noted above, research on the specific population of adolescent athletes transitioning from COVID-19 restrictions is very limited, given the narrow focus of this particular study group, and the relatively short amount of time since the height of the pandemic has ended. With this caveat in mind, though, we can learn from existing studies on aspects of this population, and dependent variables of interest, in order to compare and contrast findings with this study.

Research on the general population thus far, as well as on athletes, has indicated that many people had lower physical activity levels during the pandemic compared to before the lockdown [7, 50–53]. Overall, the disruption of regular routines due to closed exercise facilities (e.g., gyms) could have certainly contributed to lower physical activity levels for everyone.

However, there may be other factors at play for athletes. As detailed above, the participants in the current study were given individualized exercise programs by their coaches during lockdown that could have deliberately involved less activity than pre-pandemic levels (e.g., team athletes could not run drills with others, so the focus was more on strength and conditioning training). It is also possible that athletes may have injured themselves during the restriction period, which could have prevented them from engaging in higher levels of physical activity than desired. Similarly, athletes may also have a fear of potentially injuring themselves upon their return to sports, which could hold them back from deciding to reengage. Prior research by Woods et al. have indicated that during similar times of transition (e.g., athletes have stepped away from their sport for any number of reasons, and are now faced with a choice as to whether they will return or not), factors including athlete burnout, perceived stress, physical and emotional exhaustion, reduced sense of accomplishment, sport devaluation, and negative emotions about their return to sports, could prevent them from resuming their sports [52].

Even without the added stressors of major transitions, particularly during the significant “change event” of COVID-19, previous work by Marcus et al. [13], as well as others, reaffirms that it is very difficult to change activity levels. Indeed, in different populations across different time periods, they have found that in studies of exercise adoption and maintenance, many of the participants did not change their “stable” activity levels (whether “stable sedentary” or “stable active”) over the follow-up [13]. Our results of “stable” physical activity in the athletes (whether relatively low or high, depending upon factors noted above) is thus consistent with prior studies. However, to the best of our knowledge, ours is the first study to examine stages of physical activity changes specifically in an adolescent athlete population, and during a “change event.”

Our correlation between physical activity levels and mental health concerns (e.g., somatic symptoms, anxiety/insomnia, social dysfunction, severe depression) also align with the findings of several other COVID-19 studies [7, 9, 11]. In their study, Denerel et al. noted that 42.8% of their adolescent athlete participants reported feeling depressed while they were quarantined in their homes [8]. Woods and colleagues indicated that the majority of their adolescent athlete participants identified with theme of “missing the team environment” as a negative aspect of sports suspension [52]. These results underscore the key role that social contact (or lack thereof) plays in affecting overall mental health, particularly among adolescent athletes (who are used to social contact with their teammates and coaches in their regular sports routines).

The association of eating disorder symptoms (e.g., dieting, preoccupation with food, impulses to binge and purge, attempts to control food intake) with physical activity levels and mental health concerns in our study is consistent with the findings of Buckley et al. [12] during the beginning of the pandemic. As noted above, current and former athletes reported disordered body preoccupation, inhibitory food control, fear of body composition changes, and binge eating. It is possible that, with the closure of athletic facilities, more restricted exercise routines, and decreased physical activity, athletes may have experienced a loss of muscle fitness, and/or gained weight. This may motivate an athlete to increase their physical activity to an excessive level to compensate (as is often seen in eating disorders), however, this in turn reinforces the eating disorder symptoms in a vicious cycle. It is also conceivable that eating disorder symptoms may have prevented the athletes from returning to their sport (e.g., decreased energy from lack of proper nutrition, damage to organs from binging and purging). However, over time, continued eating disorder thoughts and behaviors lower overall mental health, which may bring adolescent athletes back to the “decision balance” between either increasing or decreasing their physical activity levels. Again, the athlete must consider all of the factors in making a final determination.

The role of emotional intelligence as a coping skill to alleviate mental health concerns and eating disorder symptoms corresponds with previous studies in other populations. Davis and Humphrey noted that adolescents with higher emotional intelligence had less mental health diagnoses, and fewer suicide attempts [18]. In a meta-analysis, Zhang et al. reported participants with higher emotional intelligence were less likely to have an eating disorder diagnosis [19]. However, to the best of our knowledge, this is the first study in an adolescent athlete population during a time of major transition to indicate that emotional intelligence may serve as a viable coping mechanism to address mental health concerns, and specific eating disorder symptoms.

### Clinical implications

In order to address the clinical implications of physical activity and behavioral change motivation levels, mental health concerns, and eating disorder symptoms for sports professionals working with athletes, Buckley et al. [12] suggest some key Practice Principles. These include: (1) Control (channeling control away from restrictive diets and excessive); (2) Variety (having a range of food and exercise); (3) Adaptability (being able to adapt to altered appetite needs); (4) Support (finding people that can assist with challenges); (5) Connection (connecting to one’s changing needs through intuitive eating/movement); and (6) Acceptance (accepting that the body is not infinitely malleable, and seeking non-judgmental acceptance through times of transitions).

For emotional intelligence skill development, several training programs have been created to address this need in different formats. For example, Park and Jeon [54] conducted a bibliometric analysis of 405 articles in between 1992 and 2021 related to Psychological Skills Training (PST) for athletes. Thematic elements were grouped into four clusters: (1) PST for stress, mental toughness, and coping; (2) PST for anxiety, motivation, self-confidence, and self-efficacy; (3) PST for flow and mindfulness; and (4) PST for emotions. The authors concluded that the field of PST is converging toward best performance with stress management, anxiety control, and coping skills [54].

Overall, the findings from the current study highlight the need for sports professionals working with adolescent athletes transitioning from COVID-19 to monitor these aspects of mental, eating, and emotional health. Given that those in earlier motivation stages had more health concerns compared to those in later stages, professionals should encourage progression from the Pre-Contemplation to Action/Maintenance stage in order to improve health outcomes.

### Limitations

The findings of the current study must be viewed within the context of certain limitations.

First, the study utilized a sample of Iranian adolescent athletes after the COVID-19 quarantine sports restrictions were lifted in that area (January 2022–January 2023); therefore, results may not generalize to other populations. Future research should endeavor to conduct similar studies in other populations, in order to compare, and potentially replicate, the results. Second, the participant self-report measures were not supported by additional investigator conducted diagnostic interviews, which could lead to under-reporting of related concerns. Subsequent research should endeavor to also include investigator interviews (e.g., in person, or by tele-interview) to support the self-report measures. Finally, while our findings indicate “stable” (maintained levels of) physical activity, overall mental health concerns, and eating disorder symptoms continuing from the lockdown restrictions, subsequent research should employ a longitudinal design to track any changes in these variables (e.g. in future times of “transitions”), as well as to evaluate the effect of emotional intelligence coping skills in mitigating the risk of long-term (physical/mental) health consequences.

## Conclusions

In this sample of adolescent athletes in the process of transitioning back to their sports once the COVID-19 area restrictions were lifted, there were significant differences between physical activity and related stages of motivation for behavioral change participant groups. Notably, participants in the Pre-Contemplation group (not intending to make behavioral changes within the next 6 months) had less mental health, higher eating pathology, and lower emotional intelligence, compared to participants who were in the Preparation group (intending to make behavioral changes within the next 1 month). Similarly, participants in the Preparation group had less mental health, higher eating pathology, and lower emotional intelligence, compared to participants who were in the Action group (has made specific, overt behavioral changes within the preceding 6 months). Thus, participants who were in an earlier motivation stage had more health concerns compared to participants who were in a later motivation stage (Pre-Contemplation vs. Preparation, Preparation vs. Action).

Clinical implications for these results include the improvement of skills related to control, variety, adaptability, support, connection, and acceptance, which may improve the relationships between athletes and these variables [55]. Specific programs targeting stress management, anxiety control, and coping skills may hold particular promise [54]. Overall, the findings from the current study highlight the need for sports professionals working with adolescent athletes transitioning from COVID-19 to monitor these aspects of mental, eating, and emotional health. Given that those in earlier motivation stages had more health concerns compared to those in later stages, professionals should encourage progression from the Pre-Contemplation to Action/Maintenance stage in order to improve health outcomes.

### Supplementary Information

**Additional file 1. Supplemental Table 1.** Frequency and Percentage of Eating Disorder Symptoms Subscale Clinical Cut-Off Scores by Physical Activity and Behavioral Change Motivation Groups.

**Additional file 2. Supplemental Table 2.** Differences Between Physical Activity and Behavioral Change Motivation Groups on Measures of Mental Health Concerns, Eating Disorder Symptoms, and Emotional Intelligence.

## Data Availability

The datasets generated and analyzed during the current study are not publicly available, as individual privacy could be compromised, but are available from the corresponding author on reasonable request.

## Abbreviations

EAT-26: Eating Attitudes Test-26
GHQ-28: General Health Questionnaire-28
LSD: Least square difference
MANOVA: Multiple analysis of variance
PAR: Physical activity recall
PASCQ: Physical activity stages of change questionnaire
SSEIT: Schutte Self-Report Emotional Intelligence Test
SD: Standard deviation
SPSS: Statistical package for social science
TTM: Transtheoretical model
