# Supplementary material for: Associations between physical activity, mental health concerns, eating disorder symptoms, and emotional intelligence in adolescent athletes transitioning from COVID-19
Source: J Eat Disord. 2024 Jan 2;12:2. doi: 10.1186/s40337-023-00961-2 (PMC10759499; doi:10.1186/s40337-023-00961-2)
Supplement: Supplementary file 1 — Additional file 1. Supplemental Table 1. Frequency and Percentage of Eating Disorder Symptoms Subscale Clinical Cut-Off Scores by Physical Activity and Behavioral Change Motivation Groups. [file 40337_2023_961_MOESM1_ESM.docx]

**Supplemental Table 1. Frequency and Percentage of Eating Disorder Symptoms Subscale Clinical Cut-Off Scores by Physical Activity and Behavioral Change Motivation Groups**

| Eating Disorder Symptoms Subscale  Physical Activity and Behavioral Change Motivation Groups | Dieting | | Bulimia and Food Preoccupation | | Oral Control | | Below Clinical  Cut-Offs | |
| --- | --- | --- | --- | --- | --- | --- | --- | --- |
|  | Frequency | Percentage | Frequency | Percentage | Frequency | Percentage | Frequency | Percentage |
| Pre-Contemplation | 16 | 5.07 | 17 | 5.39 | 17 | 5.39 | 35 | 11.11 |
| Contemplation | 14 | 4.44 | 11 | 3.49 | 14 | 4.44 | 33 | 10.47 |
| Preparation | 12 | 3.80 | 9 | 2.85 | 12 | 3.80 | 29 | 9.20 |
| Action | 11 | 3.49 | 8 | 2.53 | 9 | 2.85 | 23 | 7.30 |
| Maintenance | 10 | 3.17 | 8 | 2.53 | 8 | 2.53 | 19 | 6.03 |
| Total | 63 | 19.98 | 53 | 16.79 | 60 | 19.01 | 139 | 44.22 |

Physical activity and behavioral change motivation was assessed by the Physical Activity Stages of Change Questionnaire (PASCQ) (13); Eating disorders symptoms were assessed by the Eating Attitudes Test-26 (EAT-26) (40).
