# Supplementary material for: Associations between physical activity, mental health concerns, eating disorder symptoms, and emotional intelligence in adolescent athletes transitioning from COVID-19
Source: J Eat Disord. 2024 Jan 2;12:2. doi: 10.1186/s40337-023-00961-2 (PMC10759499; doi:10.1186/s40337-023-00961-2)
Supplement: Supplementary file 2 — Additional file 2. Supplemental Table 2. Differences Between Physical Activity and Behavioral Change Motivation Groups on Measures of Mental Health Concerns, Eating Disorder Symptoms, and Emotional Intelligence. [file 40337_2023_961_MOESM2_ESM.docx]

**Supplemental Table 2. Differences Between Physical Activity and Behavioral Change Motivation Groups on Measures of Mental Health Concerns, Eating Disorder Symptoms, and Emotional Intelligence**

| Variable |  | Sum of Squares | df | Mean Square | F | *p-value* |
| --- | --- | --- | --- | --- | --- | --- |
| Mental Health | Between Groups | 3,547.4 | 4 | 886.85 | 10.00 | **.001**** |
|  | Within Groups | 31,748.67 | 358 | 88.68 |  |  |
|  | Total | 35,296.07 | 362 | 975.53 |  |  |
| Eating Disorder Symptoms | Between Groups | 2,766.5 | 4 | 691.625 | 11.66 | **.001**** |
|  | Within Groups | 21,236.52 | 358 | 59.31 |  |  |
|  | Total | 24,003.02 | 362 | 750.935 |  |  |
| Emotional Intelligence | Between Groups | 2,954.4 | 4 | 738.6 | 7.88 | **.001**** |
|  | Within Groups | 33,546.17 | 358 | 93.70 |  |  |
|  | Total | 36,500.57 | 362 | 832.3 |  |  |

**** p < .01**

df = Degrees of Freedom; Physical activity and behavioral change motivation was assessed by the Physical Activity Stages of Change Questionnaire (PASCQ) (13); Mental health concerns were assessed by the General Health Questionnaire (GHQ-28) (32, 33); Eating disorders symptoms were assessed by the Eating Attitudes Test-26 (EAT-26) (40); Emotional intelligence was assessed by the Schutte Emotional Intelligence Questionnaire (SSEIT) (45).
